# The complete chloroplast genome of *Oxytropis ochrocephala* Bunge 1874 (Fabaceae) and its phylogenetic analysis

**DOI:** 10.1080/23802359.2024.2350626

**Published:** 2024-05-17

**Authors:** XiaYu Hu, YuPing Liu, JinYuan Chen, Xu Su

**Affiliations:** a School of Life Sciences, Qinghai Normal University, Xining, China; b Key Laboratory of Biodiversity Formation Mechanism and Comprehensive Utilization of the Qinghai-Tibet Plateau in Qinghai Province, Qinghai Normal University, Xining, China; c Academy of Plateau Science and Sustainability, Qinghai Normal University, Xining, China

**Keywords:** *Oxytropis ochrocephala*, chloroplast genome, IR loss, phylogenetic analysis

## Abstract

This study presents the first-ever complete chloroplast (cp) genome sequence of *Oxytropis ochrocephala* Bunge 1874, a member of the Fabaceae family. The cp genome spans 126,996 base pairs and includes 109 genes, comprising 76 protein-coding genes, 29 tRNA genes, and four rRNA genes. Notably, the genome lacks an inverted repeat (IR) region. Additionally, we constructed phylogenetic trees for 34 species within Trib. Galegeae, employing both maximum likelihood (ML) and Bayesian inference (BI) methods. These analyses robustly support the monophyly of the *Oxytropis* species, evidenced by high bootstrap values (BP = 100) and posterior probabilities (PP = 1). Within this clade, *O. ochrocephala* exhibits a sister relationship with other *Oxytropis* species. Our findings provide valuable insights into the genetic makeup and evolutionary relationships of *O. ochrocephala* within the Galegeae tribe.

## Introduction

*Oxytropis ochrocephala* Bunge 1874, a perennial medicinal herb (Zhou et al. 2020; Xue et al. 2022; Yu 2022) within the *Oxytropis* genus of the Galegeae tribe in the Fabaceae family, exhibits notable ecological and nutritional significance. Characterized by a robust root system and strong reproductive capacity, this species demonstrates remarkable resilience to environmental stressors such as cold, drought, salinity, alkalinity, and sand winds. This resilience contributes positively to the restoration of fragile ecosystems. Nutritional analyses reveal that *O. ochrocephala*’s crude fiber and protein content are comparable to that of *Medicago sativa*, suggesting its potential as a high-quality forage resource for livestock following appropriate detoxification (Shen and Mo 2013).

Previous research on *O. ochrocephala* has primarily focused on aspects like seed germination (Chen et al. 2017), chemical composition (Zhou et al. 2020), pharmacological activity (Tan et al. 2016), genetic structure (He et al. 2015), and endophytic bacterial diversity (Jiang et al. 2019; Zhang et al. 2022). However, there is a noticeable gap in the literature regarding the structural characteristics and phylogeny of its complete chloroplast (cp) genome. The scarcity of cp genome resources among the numerous *Oxytropis* species further limits related studies.

Addressing this gap, our study involves sequencing and analyzing the cp genome of *O. ochrocephala* using advanced high-throughput sequencing technology and bioinformatics methods. Our research elucidates the cp genome structure and clarifies the phylogenetic relationships within the *Oxytropis* genus. This study aims to provide a scientific foundation for species identification and understanding the phylogenetic relationships within *Oxytropis*.

## Materials

**Figure 1.**
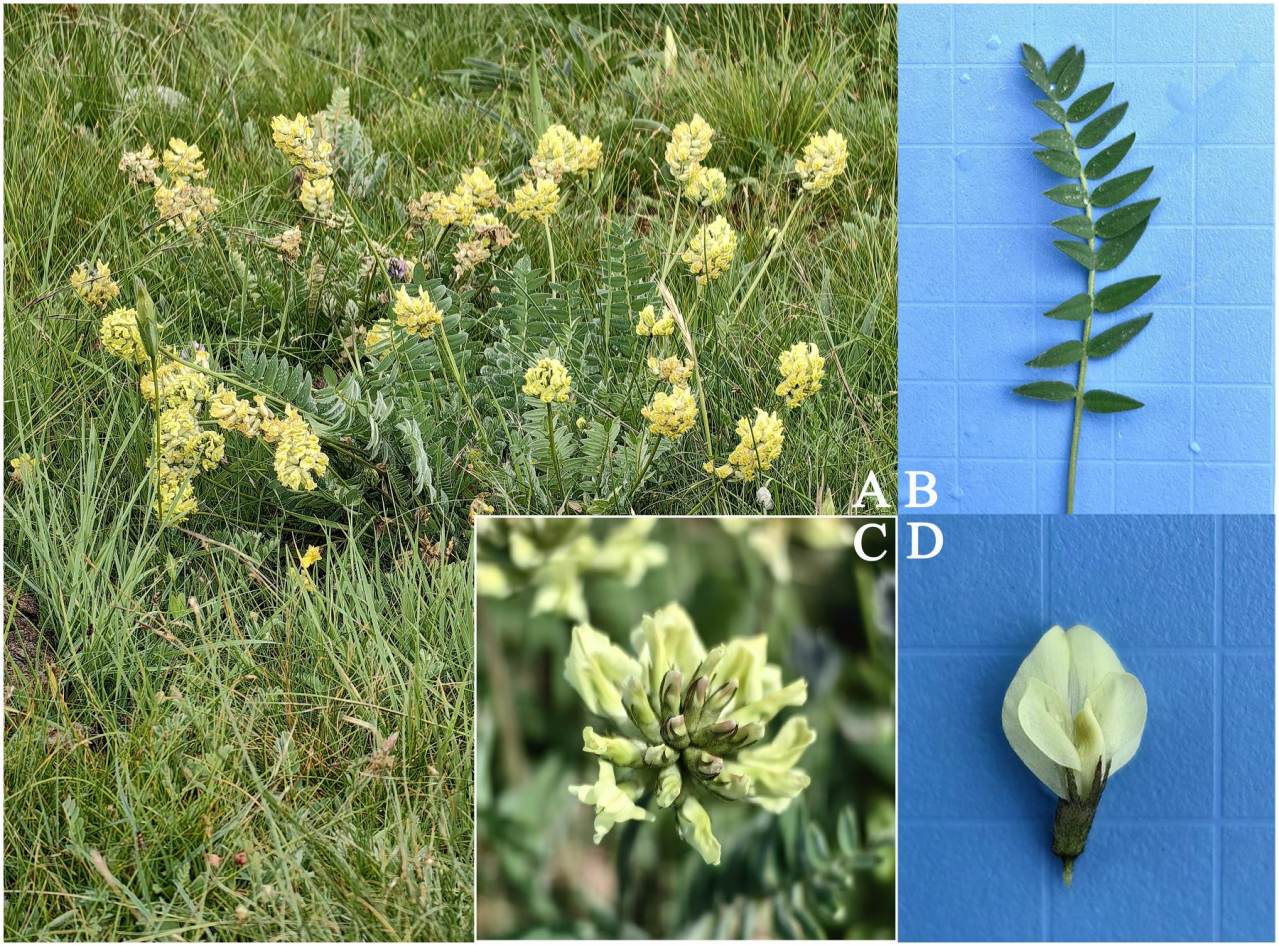
Field photos of *Oxytropis ochrocephala*. (A) Whole plant. (B) Leaf. (C) The inflorescence. (D) Flower. The author Jinyuan Chen shot the photo at the position of 37.37°N, 97.37°E. Main identifying traits: stems erect or sprawling, with 2–5 or more apparent internodes, yellowish pilose. Leaves 3–19 cm, 13–27 foliolate; leaflet blades ovate-lanceolate, both surfaces strigose with sparse short or long trichomes. Racemes compact, 8–14-flowered or more; bracts linear-lanceolate, with dense trichomes. Calyx cylindrical, membranous, with dense trichomes; lobes linear-lanceolate, Corolla yellow.

For our study, we selectively collected natural, healthy, and pest-free leaves of *O. ochrocephala* from Delingha City, located in the Haixi Mongolian and Tibetan Autonomous Prefecture of Qinghai Province, China. The specific coordinates of our collection site were 37.37°N, 97.37°E, at an altitude of 2952 m (Figure 1). To ensure the preservation of the samples’ integrity, the leaves were immediately placed into silica gel for desiccation. The specimen was deposited at Qinghai Provincial Key Laboratory of Physical Geography and Environmental Processes of Qinghai Normal University, Xining, China under the voucher number QTP-LJQ-CHNQ-024-4013 (contact: Xu Su, xusu8527972@126.com).

## Methods

Total genomic DNA was extracted from the collected samples using a modified CTAB method (Doyle and Doyle 1987). To assess DNA integrity, concentration, and purity, we employed 1.0% agarose gel electrophoresis and a Nanodrop 2000 spectrophotometer, respectively. Following quality assurance, DNA meeting our criteria was utilized for library construction via PCR amplification. The library was then sequenced using next-generation sequencing (NGS) on the Illumina NovaSeq 6000 platform at Beijing Novogene Technology Company Limited, Beijing, China.

This sequencing process yielded 17,618,422 raw reads. Using Fastp software (Chen et al. 2018), we filtered these to obtain clean reads. The cp genome sequence of *O. ochrocephala* was assembled from these clean reads using GetOrganelle software with default parameters (Jin et al. 2020). Annotation of the assembled genome was performed using GeSeq (https://chlorobox.mpimp-golm.mpg.de/geseq.html) (Tillich et al. 2017) and CPGAVAS2 (http://47.96.249.172:16019/analyzer/home) (Shi et al. 2019), referencing the *O. glabra* (MW349014) genome. Discrepancies between annotations from the two software tools were reconciled by removing redundant and incorrect information. The gene map of *O. ochrocephala’*s cp genome was visualized using CPGview (CPGview, http://47.96.249.172:16085/cpgview/home) (Liu et al. 2023). The finalized cp sequence and annotation information were submitted to GenBank (accession number OR897029).

**Figure 2.**
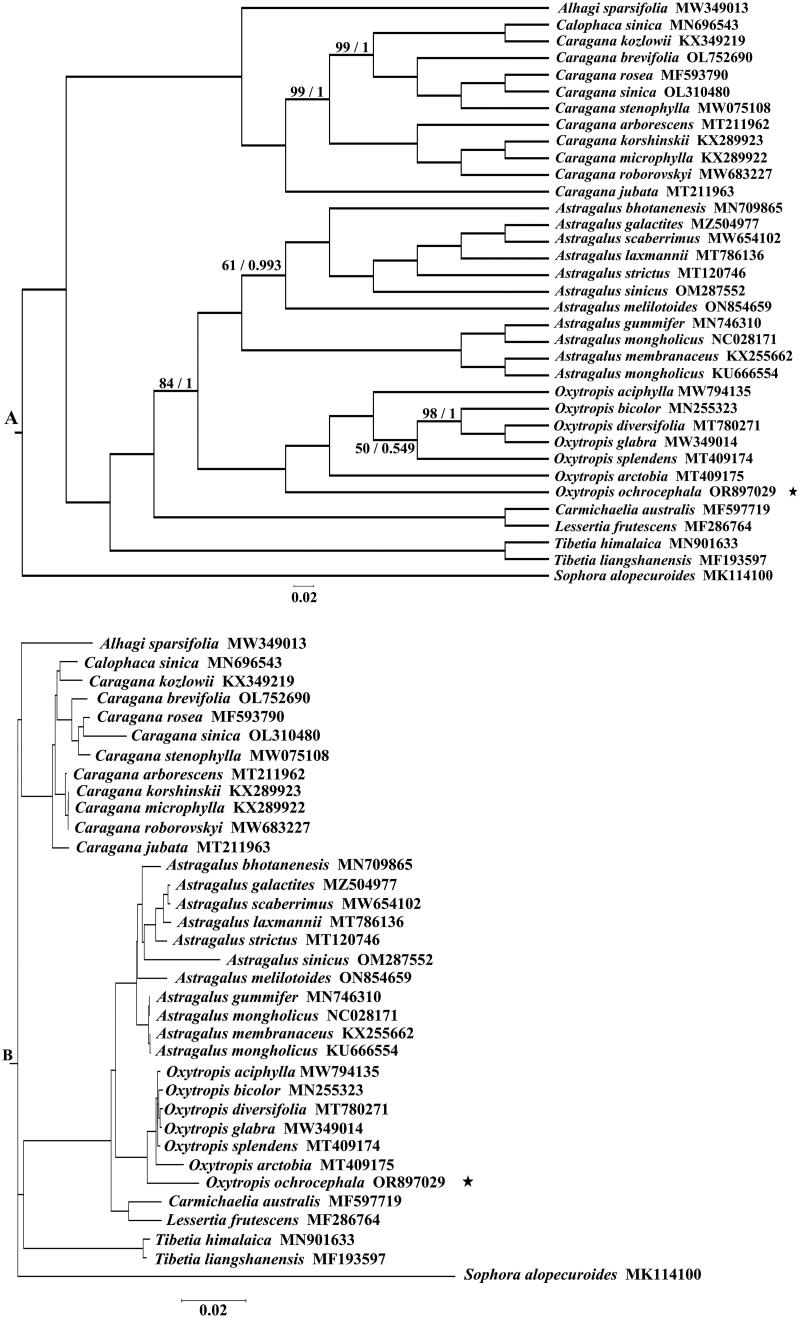
Phylogenetic tree reconstructed by ML and BI analysis based on cp genome sequences of 34 species of Trib. Galegeae. Bootstrap values from the ML and BI analysis are shown above branches (ML/BI), and the bootstrap values of 100/1 are not shown in panel A. The corresponding phylogram tree is shown in panel B. Species in this study are marked with ⋆ in A and B. We downloaded 34 Trib. Galegeae species plastomes from GenBank, *Alhagi sparsifolia* (MW349013) (Jiang et al. 2021), *Calophaca sinica* (MN696543) (Guo et al. 2020), *Caragana brevifolia* (OL752690), *C. rosea* (MF593790) (Jiang et al. 2018), *C. sinica* (OL310480), *C*. stenophylla (MW075108), *C. arborescens* (MT211962) (Liu et al. 2023), *C. korshinskii* (KX289923), *C. microphylla* (KX289922) (Liu et al. 2016), *C. roborovskyi* (MW683227), *C. jubata* (MT211963), *Astragalus bhotanensis* (MN709865), *A. galactites* (MZ504977) and *A. strictus* (MT120746) (Ding et al. 2021), *A. scaberrimus* (MW654102) (Guo and Wariss 2021; Guo, Wariss, et al. 2021; Guo, Yao, et al. 2021), *A. laxmannii* (MT786136) (Liu et al. 2020), *A. sinicus* (OM287552) (Ke et al. 2022), *A. melilotoides* (ON854659) and *A. gummifer* (MN746310) (Wang et al. 2023), *A. mongholicus* (NC028171) (Choi et al. 2016), *A. membranaceus* (KX255662) (Wang et al. 2016), *A. mongholicus* (KU666554), *O. mongholicus* (KU666554), *O. aciphylla* (MW794135), *O. bicolor* (MN255323) (Su et al. 2019), *O. diversifolia* (MT780271), *O. glabra* (MW349014) (Liu et al. 2021), *O. splendens* (MT409174) and *O. arctobia* (MT409175) (Juan et al. 2022), *Carmichaelia australis* (MF597719), *Lessertia frutescens* (MF286764) (Guo and Wariss 2021; Guo, Wariss, et al. 2021; Guo, Yao, et al. 2021), *Tibetia himalaica* (MN901633), *T. liangshanensis* (MF193597) (Guo and Wariss 2021; Guo, Wariss, et al. 2021; Guo, Yao, et al. 2021), and *Sophora alopecuroides* (MK114100) (Zha et al. 2020) from Trib. Sophoreae, served as the outgroup.

Furthermore, 34 cp genome sequences of species from Trib. Galegeae, as referenced in Figure 2 with GenBank accession numbers, were aligned using MAFFT (Katoh et al. 2002). The aligned sequences were refined using trimAl (Salvador et al. 2009). Phylogenetic trees involving these 34 species were constructed, using *Sophora alopecuroides* (MK114100) as an outgroup. The maximum-likelihood (ML) trees were generated via IQ-TREE (Nguyen et al. 2015), selecting TVM + F + R4 as the optimal model and setting the bootstrap value to 5000. The Bayesian inference (BI) tree was built using MrBayes (Ronquist et al. 2012) with the GTR + F + I + G4 model.

## Results and discussion

**Figure 3.**
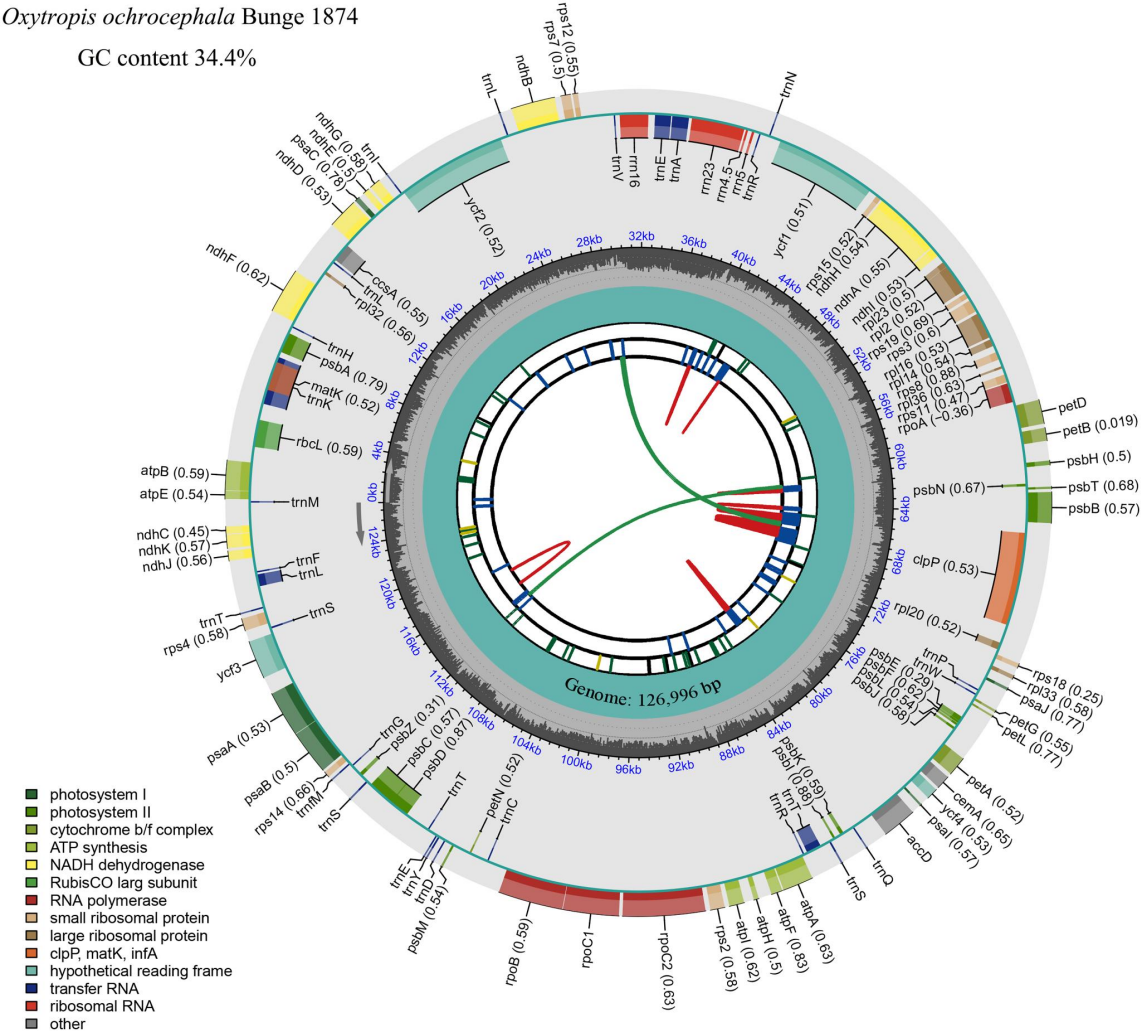
Gene map of the cp genome of *O. ochrocephala*. The species name and GC content are shown in the top left corner. The map contains six tracks in default. From the center outward, the first track shows the dispersed repeats. The dispersed repeats consist of direct and palindromic repeats, connected with red and green arcs. The second track shows the long tandem repeats as short blue bars. The third track shows the short tandem repeats or microsatellite sequences as short bars with different colors. These colors correspond to the type and description of each repeat, with black representing complex repeats, green for repeat unit size 1 and yellow for size 2. The fourth track displays the genome length. The fifth track shows the GC content along the genome, while the sixth track sounds the genes. The gene names are followed by optional information about codon usage bias and color-coded based on their functional classification. The inner genes are transcribed clockwise, and the outer genes are transcribed anticlockwise. The functional type of the genes is shown in the bottom left corner.

The 17,532,592 clean reads obtained after sequencing and quality control were located on the cp genome sequence of *Oxytropis glabra* (MW349014), and its coverage depth is shown in Figure S1. Generally, the cp genome of higher plants is 107–218 kb in length (Fan and Guo 2022), with 110–130 coding genes (Jansen et al. 2005), and the total GC content is 30–45% (Zhu et al. 2017) and GC content was different at different locations, where IR region was greater than large single-copy (LSC) region than small single-copy (SSC) region (Cai et al. 2006). The results of this study showed that the sequenced cp genome of *Oxytropis ochrocephala* spans 126,996 base pairs and exhibits an average GC content of 34.3%. This genome encodes a total of 109 genes, including 76 protein-coding genes, 29 tRNA genes, and four rRNA genes, as illustrated in Figure 3, which is basically consistent with the cp genome characteristics of other species of Oxytropis (Su et al. 2019; Liu et al. 2021). In summary, the cp genome of *O. ochrocephala* accords with the above characteristics of higher plants, but all of them are at a low level. Notably, in the cp coding genes of *O. ochrocephala*, 14 possess a single intron (specifically, *trn*A-UGC, *trn*E-UUC, *trn*K-UUU, *trn*L-UAA, *trn*T-CGU, *acc*D, *clp*P, *ndh*A, *ndh*B, *pet*D, *rpl*2, *rpl*16, *rpo*C1, and *rps*12), and the *ycf*3 gene is characterized by two introns. It also includes nine cis-splicing genes, including *ndh*B, *ndh*A, *rpl*2, *rpl*16, *pet*D, *clp*P, *acc*D, *rpo*C1, and *ycf*3, whose gene structure diagram is shown in Figure S3.

Typically, the cp genomes of angiosperms exhibit a quadripartite structure, consisting of a pair of inverted repeats (IRs), a LSC region, and a SSC region (Xu et al. 2023). In addition, the IR region plays an important role in stabilizing the structure of the cp genome, and the reduction, expansion and loss of the reverse repeat region directly affect the change of the cp genome structure (Song et al. 2017). However, the cp genome of *O. ochrocephala* deviates from this norm because it lacks quadripartite structure due to the absence of IR region, which may be the main reason for its shorter cp genome length, lower GC content and lower number of coding genes. Meanwhile, the characteristic absence of an IR region has also been observed in other cp genomes of *Oxytropis* genus (Su et al. 2019; Liu et al. 2021). This leads to the hypothesis that the loss of an IR region may be a common feature of the cp genomes in the *Oxytropis* genus. It may also be related to the process of species differentiation of *Oxytropis*.

Utilizing the cp genome sequences of 34 species from Trib. Galegeae, we constructed phylogenetic trees using both ML and BI methods, with *Sophora alopecuroides* serving as the outgroup for each analysis. The resultant phylogenetic trees, as illustrated in Figure 2(A), demonstrated identical topologies with high support values (100/1) across the majority of branches. Notably, the *Oxytropis* genus formed a monophyletic group in both trees. Within this group, *O. ochrocephala* displayed a sister relationship to the other species in the *Oxytropis* genus, as depicted in Figure 2(B).

## Conclusions

This study successfully sequenced the cp genome of *O. ochrocephala* for the first time, revealing a genome length of 126,996 base pairs and an average GC content of 34.3%. The genome comprises 109 genes, which include 76 protein-coding genes, 29 tRNA genes, and four rRNA genes. Notably, the cp genome exhibits a unique circular structure, distinguished by the absence of an IR region. Phylogenetic analysis based on this genome sequence indicates that *O. ochrocephala* shares a sister relationship with other species within the *Oxytropis* genus.

## Supplementary Material

Supplemental Material

## Data Availability

The complete chloroplast genome sequence of *O. ochrocephala* in this study has been submitted to the NCBI database under the accession number OR897029. The associated BioProject, BioSample, and SRA numbers are PRJNA1054665, SAMN38930174, and SRR27321281, respectively.
