# Supplementary material for: The complete chloroplast genome of Oxytropis ochrocephala Bunge 1874 (Fabaceae) and its phylogenetic analysis
Source: Mitochondrial DNA B Resour. 2024 May 17;9(5):641–6. doi: 10.1080/23802359.2024.2350626 (PMC11104710; doi:10.1080/23802359.2024.2350626)
Supplement: Supplemental Material [file TMDN_A_2350626_SM3360.docx]

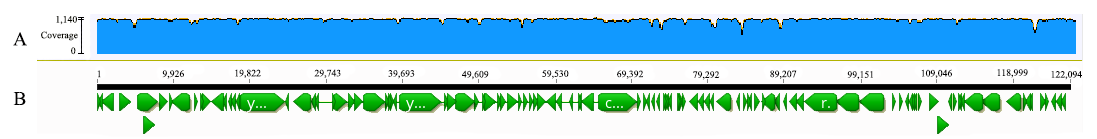


Figure S1. Depth of coverage plot (A) and physical map of cpDNA (B). The 17,532,592 clean reads from Illumina sequencing were remapped on the cp genome sequence of *Oxytropis glabra* (MW349014) using Bowtie2 as implemented in Geneious prime 2023.


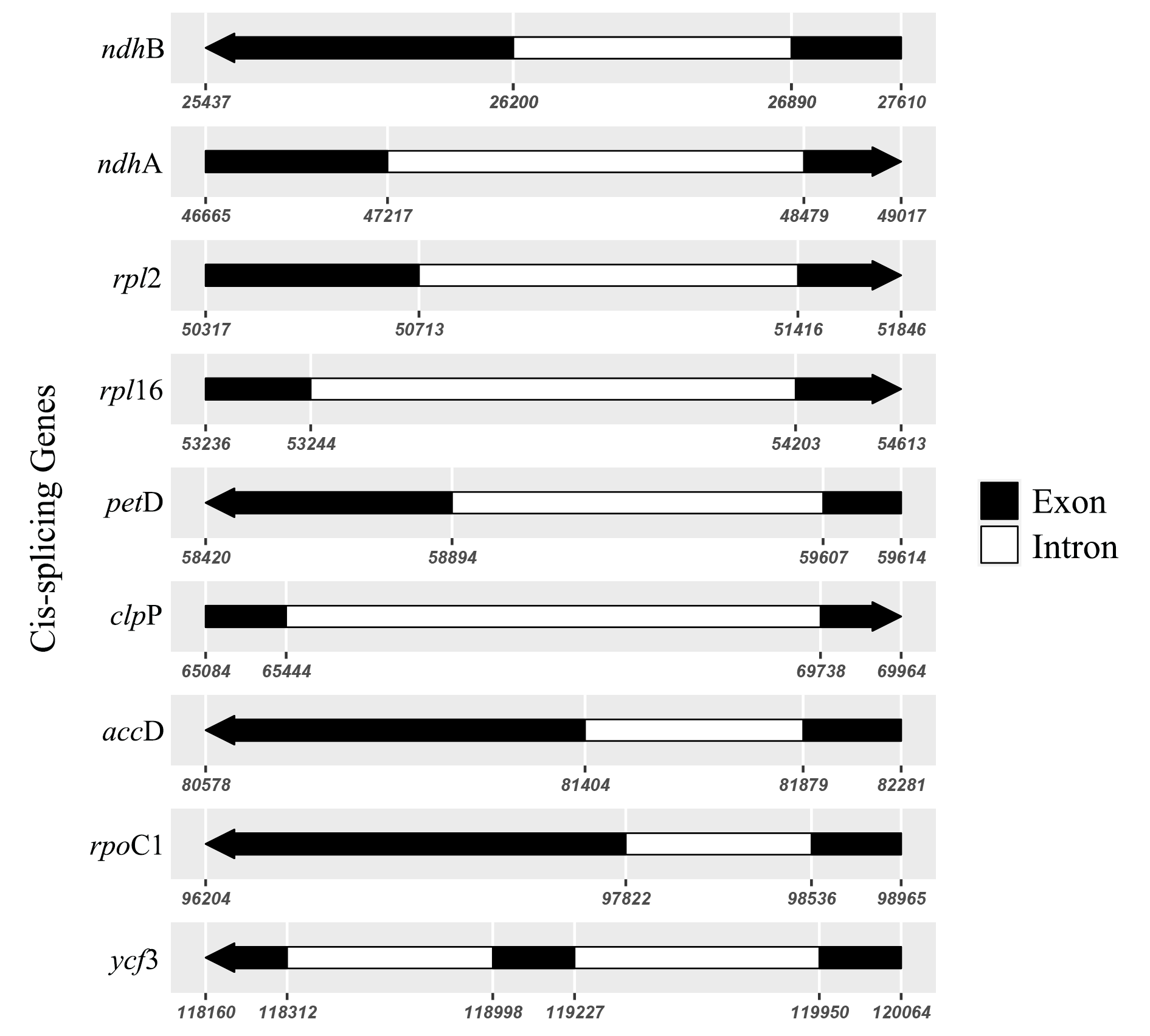


Figure S2. Schematic maps of cis-splicing genes in the chloroplast genome of *Oxytropis ochrocephala*. Maps generated using CPGView. The gene names are shown on the left, and the gene structures are on the right.
